# The fungus *Escovopsis* (*Ascomycota*: *Hypocreales*): a critical review of its biology and parasitism of attine ant colonies

**DOI:** 10.3389/ffunb.2024.1486601

**Published:** 2025-03-18

**Authors:** Simon Luke Elliot, Quimi Vidaurre Montoya, Marcela Cristina Silva Caixeta, Andre Rodrigues

**Affiliations:** ^1^ Department of Entomology, Universidade Federal de Viçosa, Viçosa, Brazil; ^2^ Department of Genetics, Evolution and Environment, Centre for Biodiversity and Environment Research, University College London, London, United Kingdom; ^3^ Department of General and Applied Biology, São Paulo State University (UNESP), Rio Claro, Brazil

**Keywords:** fungi, fungus-cultivating insects, mycoparasitism, host-parasite interactions, leafcutter ants, virulence, ecology, evolution

## Abstract

Two biological phenomena that contribute to increasing complexity in biological systems are mutualistic symbiotic interactions and the evolution of sociality. These two phenomena are also of fundamental importance to our understanding of the natural world. An organism that poses a threat to one or both of these is therefore also of great interest as it represents a challenge that mutualistic symbioses and social organisms have to overcome. This is the case with the fungus *Escovopsis* (*Ascomycota*: *Hypocreales*), which attacks the fungus garden of attine ants (*Formicidae*: *Attina*) such as the leaf cutters. This parasite has attracted much high-profile scientific interest for considerable time, and its study has been fruitful in understanding evolutionary, ecological and behavioural processes. Despite this, much of the biology and ecology of this organism remains unknown. Here we discuss this fungus and three sister genera (*Escovopsioides*, *Luteomyces* and *Sympodiorosea*) that until recently were considered as a single group. We first describe its position as the most highly specialised microbial symbiont in this system other than the mutualistic fungal cultivar itself and as that of greatest scientific interest. We then review the taxonomic history of the group and its macroevolution and biogeography. We examine what we know of its life cycle in the field – surprisingly little is known of how it is transmitted between colonies, but we explain what is known to date. We then review how it interacts with its host(s), first at the level of its direct interaction with the basidiomycete host fungi wherein we show the evidence for it being a mycoparasite; then at the colony level where empirical evidence points towards it being a parasite with a very low virulence or even merely a opportunist. Finally, we offer directions for future research.

## 1 Introduction

Symbioses are of great importance as they guide the evolutionary history of the organisms involved in them (Maynard-Smith and Szathmáry, 1995; Moran, 2006; Douglas, 2010; Chomicki et al., 2019). While symbioses may be parasitic (**Table 1**), most organisms on Earth rely on mutualistic symbioses, e.g. aphid-*Buchnera* systems (Douglas, 1998), arbuscular mycorrhizae of plants (Strullu-Derrien et al., 2018), entomopathogenic nematodes and their bacterial partners (Forst et al., 1997), mitochondria and chloroplasts in eukaryotic cells (Sagan, 1967). In insect societies, there are some symbionts that have become as important to their hosts – in obligate mutualisms – as are mitochondria to eukaryotes (Schultz, 2022; **Table 1**). This is the case with fungus-growing ants (*Formicidae*: *Myrmicinae*: *Attini*: *Attina*, the “attines”) that cultivate Basidiomycete fungi in the order *Agaricales* as their main food source (Weber, 1972; Schultz and Brady, 2008; Mehdiabadi and Schultz, 2010; Della Lucia, 2011) in what can be termed agriculture (or fungiculture, see Schultz, 2022 for a full discussion of this).

**Table 1:** Definitions and important organisms.

**Agriculture** - as practised by humans is the nutritional and economic reliance on domesticated plants and animals (Ješovnik and Schultz, 2022; Schultz, 2022). For the purpose of this text, we use “agriculture” as the nutritional reliance of ants and other insects on fungi (see **fungiculture**).
**Endophytism.** A relationship established by any organism that lives within plant tissues (de Bary, 1866). For microorganisms (predominantly fungi and bacteria), this term is usually reserved for cases in which the microorganisms do not cause damage to their hosts, distinguishing them from plant pathogens (even when such pathogens, e.g. *Fusarium*, could strictly speaking be considered to be endophytic).
***Escovopsis*.** Here, we use *Escovopsis* to refer to a diverse group of fungi formerly designated as “brown spored” in several studies. We consider *Escovopsis* as a monophyletic clade apart from the genera *Luteomyces* and *Sympodiorosea* (Montoya et al., 2021).
**Eusociality.** “True” sociality, according to the prefix ‘eu-’. This is the highest degree of social organisation and must have three characteristics: (i) cooperative brood care – parental care of young individuals by nonreproductive or less reproductive workers, (ii) reproductive division of labour – adults separated in reproductive castes, in which the workers are partially or totally nonreproductive; and (iii) overlapping generations in the colony; Wilson, 1971).
**Fungicolous fungi.** Fungi that are consistently found in association with other fungi (Barnett, 1963), irrespective of the nature of any relationship between the two. Many other terms are in use (e.g. mycophilic, hyperparasitic) with slightly different meanings but we deliberately avoid these terms and here use only fungicolous and mycoparasitic - the reader is referred to Sun et al. (2019) for a full treatment of these terms.
**Fungiculture.** The practice of cultivating fungi for food. This lifestyle evolved in three insect orders: ants and stingless bees (Hymenoptera: Formicidae and Apidae); beetles in the subfamilies Scolytinae and Platypodinae (Coleoptera: Curculionidae) (12); and termites in the subfamily Macrotermitinae (Blattodea: Termitidae) (13). In all these cases, there is a dependence on the cultivar for food (Mueller et al., 2005), although there are instances of fungiculture for materials (*versus* nutrition), much as with human agriculture (Dejean et al., 2023).
**Horizontal transmission.** Transmission of symbionts (whether these are **mutualistic**, **parasitic** etc) among individuals of the same generation (Ewald, 1994), but also across different (descendant) lines – i.e. not to own offspring. In the case of social insect colonies that can be treated as **superorganisms**, this can be applied to the transmission of symbionts between colonies that have already been founded.
**Mutualism.** An interspecific interaction in which the fitness benefits that accrue due to the interaction are greater for both partners than the respective costs. This association may be symbiotic or not and can be facultative or obligatory.
**Mycoparasitism.** Parasitism of a fungus (the mycoparasite) by another (**fungicolous**) fungus (host or mycohost) (Barnett, 1963; Sun et al., 2019). The fitness of the latter is decreased on balance (see **parasitism**).
**Mycophagy.** The consumption of fungi for nutrition.
**Parasitism.** An interaction in which a **symbiont** causes a net reduction in its host’s fitness. It is important to note that a **symbiont** whose costs to its host outweigh any benefits is by definition a parasite, even if it was once a mutualist (Bronstein, 1994). This reduction in host fitness is the parasite’s **virulence**.
**Saprotrophic.** Fungi that degrade and feed on decomposing organic matter. Also generally referred to as “saprophytic”.
**Semisociality.** These differ from eusocial organisms in the absence of overlapping generations (Wilson, 1971).
**Superorganism** The concept of a colony of social insects as an organism, originally formulated by Wheeler (1911) for ants. In this concept, a colony can be considered to behave as a unit, have characteristics that mark it as belonging to a given species yet with intraspecific variation between colonies, have an adaptive cycle of growth and reproduction and be differentiated into soma (i.e. workers) and germ plasma (reproductives) (Wilson, 1971).
**Symbiosis (symbiont).** An interspecific interaction in which two organisms live together (de Bary, 1879) for a considerable part of the lifespan* of at least one of the organisms. Despite lay interpretations, this interaction is irrespective of (one might say orthogonal to) effects of this association on either organism´s fitness (**mutualism, parasitism** etc.). **Parasitism** is therefore a form of symbiosis. In this interaction, the natural lifespan of the host is usually longer than that of its **symbiont** (excluding resting phases) while the host is also usually the larger of the two. * “A considerable part of the lifespan” is deliberately left loose in this definition, as we consider there to be a gradient along which interactions can be more or less characterised as symbiosis.
**Virulence.** The harm that parasites cause to their hosts, ultimately reducing their fitness (Frank, 1996). A strict definition would rely upon this reduction in host fitness that arises from the association, but this is often not measured, so proxies are often used in the literature. See Dieckmann et al. (2002) for a treatment of this question.
**Vertical transmission.** Transfer of **symbionts** from parents to offspring or from one generation to the next (Ewald, 1994). In the case of social insect colonies that can be treated as **superorganisms**, this can be applied to the transmission of symbionts to newly founded colonies with the dispersal of reproductives.

Bold text indicates words (or words with the same root) for which definitions are given in the table.

As with any host-symbiont association, colonies of fungus-growing ants and their fungal partners can be used as a source of nutrients or as a habitat by other microorganisms (Fisher et al., 1996; Currie et al., 1999b; Mueller et al., 2005; Little and Currie, 2007; Rodrigues et al., 2008; Barcoto et al., 2020). If these symbiotic microorganisms harm the colony (reduce their host’s fitness) while benefiting themselves (increasing their own fitness), then they can be considered parasites (**Table 1**). A considerable number of filamentous fungi can be found in association with the colonies of these insects and may potentially be antagonists of these colonies (Rodrigues et al., 2005a, b, 2008; Van Bael et al., 2009b; Rocha et al., 2014). Nevertheless, the vast majority of these fungi are usually considered to be transient in this setting, even if they are mycoparasites (**Table 1**) in other environments or transient mycoparasites. This is the case with some mycoparasitic fungi in the genera *Trichoderma* and *Hypomyces*, as well as the saprotrophic fungus *Syncephalastrum* and others, even when they have been shown experimentally to be able to cause harm to the fungus gardens or colonies (Barcoto et al., 2017; Rocha et al., 2017; Bautz et al., 2023). These fungi have conventionally been termed “weeds” in the context of the fungus gardens of attine ants (Currie et al., 1999a; Currie and Stuart, 2001; Rodrigues et al., 2008; Augustin et al., 2013), although a better term is warranted.

**Figure 1:**
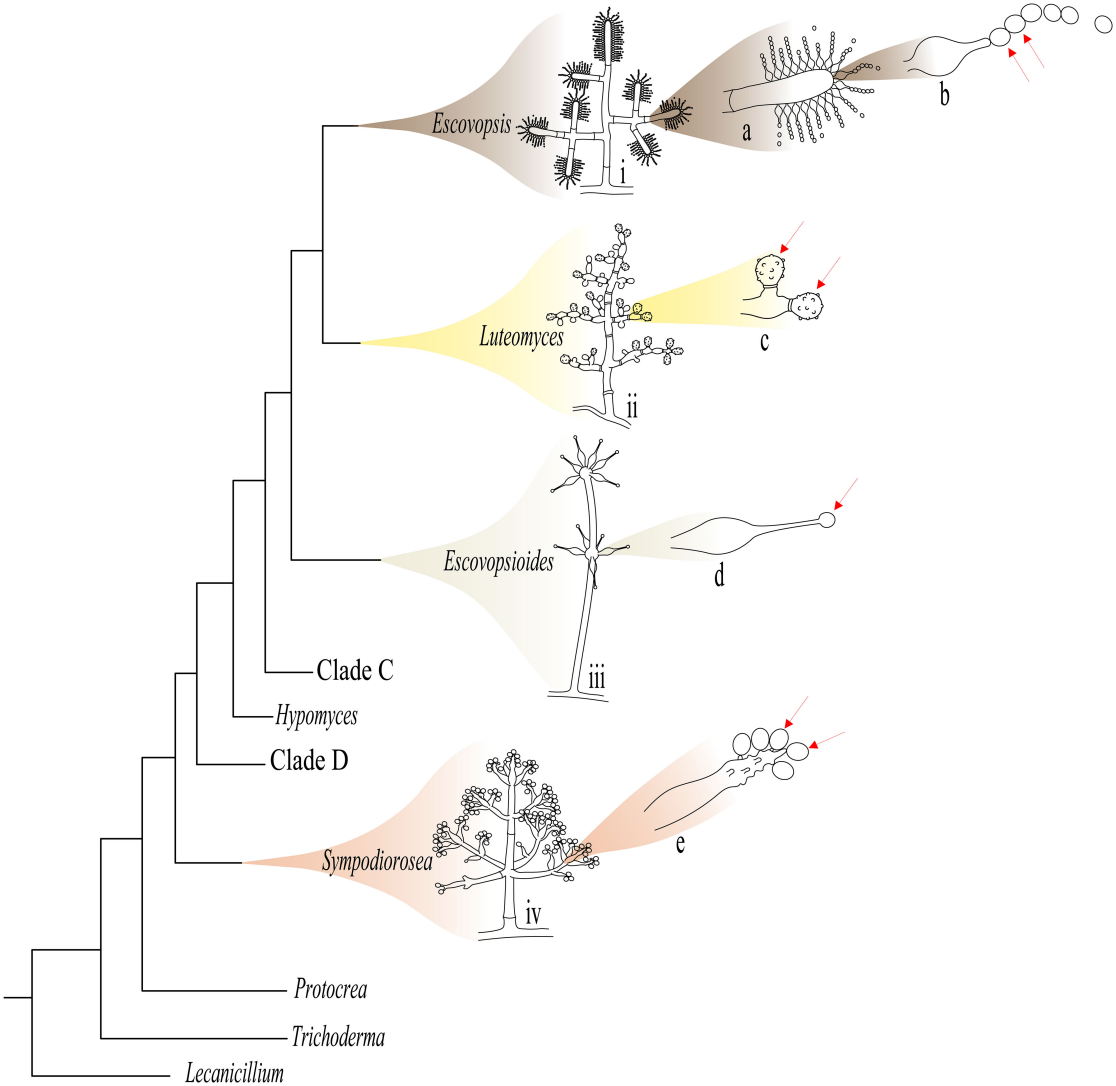
Illustrative diagram of the main microscopic morphological differences between *Escovopsis* and the other *Hypocreaceae* genera that inhabit the colonies of *Attina* ants. To the right of each genus are shown the conidiophores of: i) *Escovopsis*; ii) *Luteomyces*; iii) *Escovopsioides*; and iv) *Sympodiorosea*. To the right of each conidiophore are shown: **(A)** *Escovopsis* vesicle; **(B)** phialidic conidiogenic cell of *Escovopsis*; **(C)** indeterminate conidiogenic cell of *Luteomyces*, **(D)** phialidic conidiogenic cell of *Escovopsioides*; **(E)** sympodial conidiogenic cell of *Sympodiorosea*. Red arrows indicate the conidia of each genus. Clades C and D are also associates of colonies of *Attina* (see Montoya et al. 2021), but morphological characters of these groups of fungi are still unknown. The phylogenetic tree and the drawings of the conidiophores of *Luteomyces* and *Sympodiorosea* were modified from Montoya et al. (2021).

One fungus that is found in attine fungus gardens and has attracted particular attention is *Escovopsis*. This has generally been considered distinct from other fungi found in this environment due to an inferred ancient association with this habitat, a high degree of specialisation and a supposedly high virulence to attine colonies, that is, its parasitic habit (Currie et al., 1999a; Currie, 2001; Reynolds and Currie, 2004; Gotting et al., 2022). Over the past few decades, however, much has been learned about *Escovopsis*. Firstly, taxonomic advances have made it clear that what was long considered a single genus can now be considered to be a clade with at least four genera: *Escovopsis*, *Escovopsioides*, *Sympodiorosea* and *Luteomyces* (Augustin et al., 2013; Montoya et al., 2021; **Figure 1**). Secondly, much of the work describing the patterns of attraction and preference of *Escovopsis* to its hosts was conducted on strains now known to belong to *Sympodiorosea* (Gerardo et al., 2004, 2006a; Birnbaum and Gerardo, 2016; Custodio and Rodrigues, 2019; Montoya et al., 2021). Thirdly, the means by which this group of fungi may parasitise their hosts are still a mystery. Fourthly, almost nothing is known about the life cycle of this fungus in the field, in particular how it is transmitted between colonies. Finally, recent studies have recast this supposedly virulent parasite as a low-virulence parasite or even an opportunist (de Mendonça et al., 2021; Jiménez-Gómez et al., 2021).

**Figure 2:**
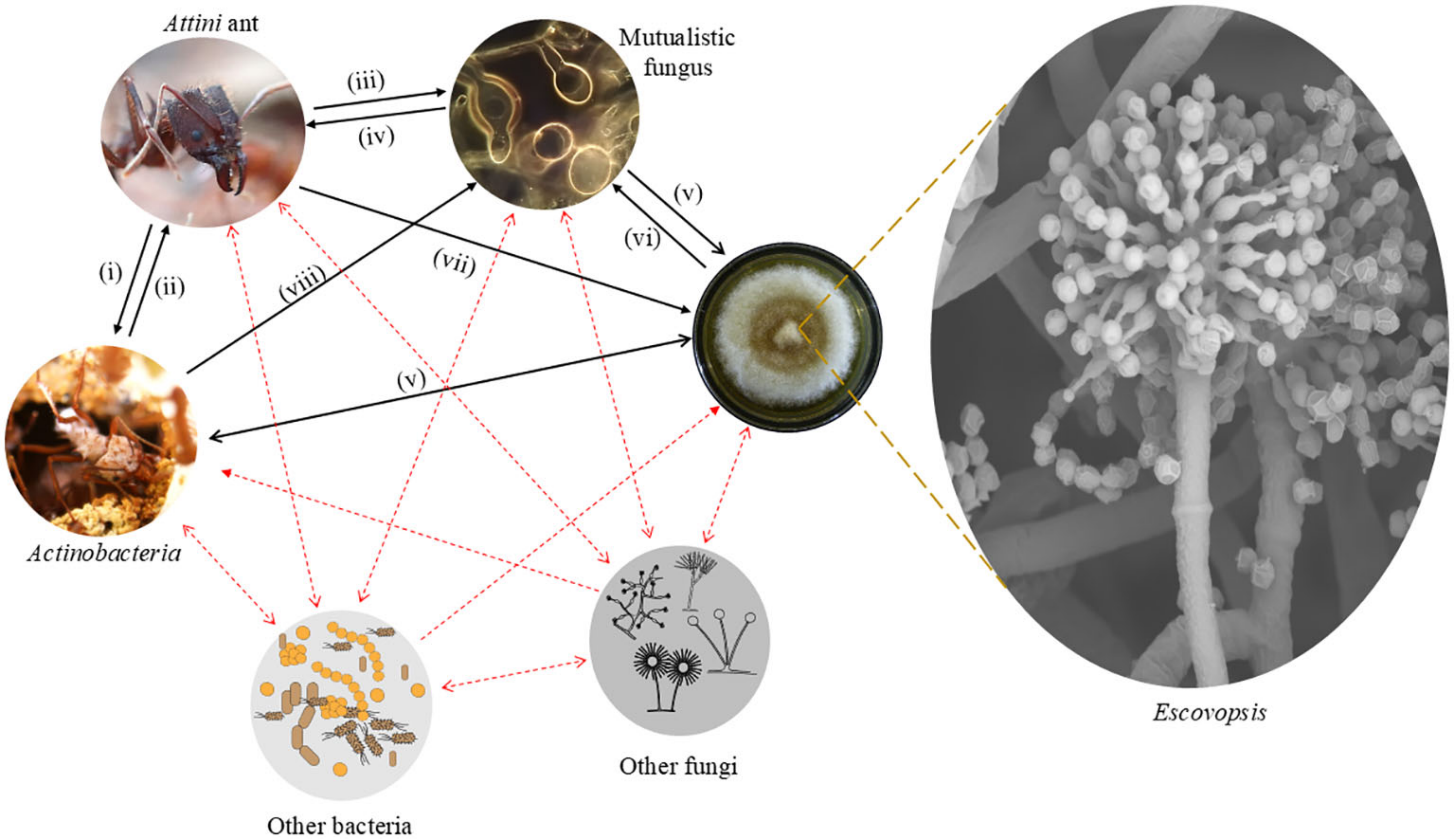
Illustrative schema of symbiotic network within colonies of *Attina* ants. The network marked with black arrows corresponds to the symbiotic relationships known to date. (i) Space; Nutrition, Dispersal; (ii) Protection; (iii) Nutrition, Protection, Dispersal; (iv) Nutrition; (v) Inhibition (possibly Competition); (vi) Threat; (vii) Weeding (i.e. removal); (viii) Protection. The network marked with red dashed arrows corresponds to the symbiotic relationships that have yet to be studied. The photos of the ant with *Actinobacteria* attached to its body and the Mutualistic fungus were provided by Enzo Roberto Sorrentino and Maria Jesus Sutta Martiarena, respectively.

Here we gather available evidence on the *Escovopsis* clade to provide a more comprehensive picture of its biology and ecological role within colonies of the fungus-growing ants. We first briefly describe fungiculture practised by insects and specifically attine ants, so as to provide some context, including an overview of other organisms that may be found in this habitat. We highlight the fact that there are countless relationships that are still unknown (**Figure 2**) and that could eventually modify our current thinking. We then focus on the *Escovopsis* clade, beginning with a historical overview, moving on to discuss taxonomic considerations for the four genera, the diversity of fungi within the clade, its geographical distribution, and then what is and is not known about its life cycle and transmission to new colonies. We continue with a discussion of the nature of the interaction of this group of fungi with the attine ant cultivars and in turn with the attines themselves. In this tour of the *Escovopsis* group, we offer critical appraisals of some areas of study, in particular discussing the evidence, considerations and ideas, raised in previous studies, that led the researchers to conclude that *Escovopsis* has a mycoparasitic lifestyle. To aid the reader, we provide some definitions of fundamental concepts and explanations of the principal players (**Table 1**). Towards the end of this review, we compare these fungi with other mycoparasitic fungi and conclude by suggesting future areas of study we hope will help researchers to unravel the evolutionary history of this charismatic group of fungi and its role in present-day neotropical ecosystems.

## 2 Fungal agriculture and fungal “weeds”

### 2.1 Fungiculture in insects

While fungi are used as a food source by many arthropods, culturing of fungi (i.e. fungiculture or agriculture - see **Table 1**) has arisen in a few groups, most notably *Hymenoptera* (*Formicidae* and *Apidae*), *Blattodea* (*Isoptera*), and *Coleoptera* (*Scolytinae* and *Platypodinae*). Although the principal coinage of this mutualism is nutritional, certainly for arthropod agriculturalists, other notable benefits to the fungal partners are protection and dispersal (Batra, 1963; Weber, 1972; Johnson et al., 1981; Biedermann and Vega, 2020). As with agriculture practised by human beings, there is an association between sociality and fungiculture in insects. Among *Coleoptera*, fungus-farmers are mainly semisocial (Beaver, 1989; Farrell et al., 2001; Harrington, 2005; Hulcr and Stelinski, 2017), but this is not always the case (Toki et al., 2012). Among *Hymenoptera*, fungiculture is principally found in one subtribe of ants (*Hymenoptera*: *Formicidae*: *Myrmicinae*: *Attini* tribe: *Attina* subtribe – Weber, 1972; Mueller et al., 2001) (note that ants are all social) but also in non-attine ants (Dejean et al., 2023), and stingless bees (*Hymenoptera*: *Apidae*: *Trigonini* tribe; Menezes et al., 2015). Curiously, fungiculture is known from non-social rather than social wasps (*Hymenoptera: Siricidae* and *Xiphydriidae*; Biedermann and Vega, 2020; Barcoto and Rodrigues, 2022). Agriculture has arisen independently in each of these groups, yet in almost all cases where nutrition is the main benefit to the agriculturalists, the fungus can be considered as an ‘external gut’ responsible for breaking down molecules such as cellulose that the insects cannot break down alone (De Siqueira et al., 1998; Silva et al., 2006; Poulsen et al., 2014; Hulcr and Stelinski, 2017). The fungal partners in these systems may also break down toxins in the plant material (Moller et al., 2011; De Fine Licht et al., 2013; Davis et al., 2019; Zhao et al., 2019) or be used as sources of essential steroid precursors for moulting hormones (Paludo et al., 2018).

When fungicultural insects establish new colonies, they usually take fungal inoculum with them (Batra, 1963; Weber, 1966; Johnson et al., 1981). Whether one considers these insect colonies as patches within metapopulations or as superorganisms, these dispersal events have much in common with the vertical transmission of symbionts from parent hosts to their offspring. As such, the term vertical transmission is widely employed in these systems. The insects usually provide excavated chambers underground or galleries in trees for their fungal partners and these sites can be at once protected and maintained as homeostatic environments (Odling-Smee et al., 2003; Biedermann and Vega, 2020).

As in any biological system, these environments can be colonised by other organisms. Probably the most abundant arthropods to do so are mites (Campbell and Crist, 2016), but it is worth noting that there may be comparatively closely related social parasites that are able to exploit these environments, certainly in the case of the attines (e.g. Sumner et al., 2003). Most fungal gardens, though, will be colonised by a wide range of other microorganisms such as bacteria, yeasts and filamentous fungi. Focusing on fungal intruders, though, the fungus cultivated by termites can be threatened by another fungus, *Pseudoxylaria* (*Ascomycota*: *Xylariales*), which uses resources the mutualist would otherwise use (Thomas, 1987; Visser et al., 2011). Meanwhile, some coleopteran systems can be threatened by the fungus *Ophiostoma minus* (*Ascomycota*: *Ophiostomatales*) that overgrows the mutualist fungus and hampers development of beetle larvae (Klepzig and Wilkens, 1997; Klepzig et al., 2004). Here we are dealing with a group of fungi that invade and potentially harm the fungal symbiont of attine fungus-cultivating ants, but first we will describe this fungiculture in more detail.

### 2.2 Agriculture (fungiculture) in attine ants

The cultivation of fungi by attine ants (*Formicidae*: *Myrmicinae*: *Attini*: *Attina*) is the prime example of agricultural practice in animals prior to humans, approximately 66 million years earlier in fact (Branstetter et al., 2017). Most ants practise what has been termed “lower agriculture” (Mueller et al., 2005; Schultz and Brady, 2008) in which they supply a basidiomycete fungus (*Basidiomycota*: *Agaricales*: *Leucocoprineae*) with organic material such as insect faeces and dead plant and invertebrate material (Hölldobler and Wilson, 1990; De Fine Licht et al., 2010). They are then able to feed their larvae on this fungus, although details of this are surprisingly hard to find in the literature. In lower agriculture, the mutualist fungus is able to live outside the ant colony (Mueller et al., 1998; Vo et al., 2009). In what is termed “higher agriculture” (Schultz and Brady, 2008), the fungal partner is limited to life within the colony and the association is more specialised (Chapela et al., 1994; Schultz and Brady, 2008; Schultz et al., 2015). This specialisation is such that the fungal partner produces nutrient-rich swellings of the hyphae, known as “gongylidia”, that can be detached by the worker ants to feed to their larval siblings (Quinlan and Cherrett, 1979; Chapela et al., 1994). A subset of species in the "higher attines" is known as leaf-cutting ants as they mainly provide fresh plant material to their fungal mutualist partners (see Hölldobler and Wilson, 1990; De Fine Licht et al., 2010).

The origin of this ant-fungus association is a subject of much debate but is beyond the remit of the present text. What is of particular importance here, however, is the key differences in some of the fungal partners cultivated by the attine ants. While all are to be found within the order *Agaricales*, two groups stand out. In one, the fungus has been replaced by a so-called “coral fungus” of the family Pterulaceae that is cultivated by the ant genus *Apterostigma* (Dentinger et al., 2009; Leal-Dutra et al., 2020). In another, the fungus, while still from the tribe *Leucocoprinae*, is cultivated in a yeast phase, to date the only example of this in the basidiomycetes associated with attine ants (Weber, 1972). Across the range of associations, there is a pattern of coevolution and even co-cladogenesis in some cases, yet with the occasional acquisition by ants of new partners (as with the coral fungi) or transmission of fungal cultivars across ant lineages (Schultz et al., 2015; Mueller et al., 2018). This issue is of importance when one considers specialised parasites of the system and the degree to which they may or may not have evolved in a co-cladogenic fashion (Gerardo et al., 2006b; Mehdiabadi and Schultz, 2010; Birnbaum and Gerardo, 2016).

The contribution of this form of agriculture to the success of this group of ants is universally recognised (Schultz, 2022). The leafcutters are often described as the dominant “herbivores” in neotropical ecosystems (Quinlan and Cherrett, 1979; Hölldobler and Wilson, 1990), and have the status of major and intransigent pests in agriculture and silviculture (Della Lucia, 2011; Della Lucia et al., 2014).

### 2.3 Microbial symbionts of attine fungus gardens

Although attine ants are presumably selected to feed, cultivate and otherwise tend only their mutualistic fungus, their fungus gardens are far from a pure culture. Many studies have documented an enormous diversity of microorganisms living in association with the mutualistic fungus (Craven et al., 1970; Fisher et al., 1996; Carreiro et al., 1997; Currie et al., 1999b; Rodrigues et al., 2005a, b, 2008, 2011; Barcoto et al., 2020). At first, most of these organisms were considered airborne contaminants (Weber, 1966, 1979) or even an asexual stage of the fungus cultivated by ants, as assumed by Möller (1893). Microorganisms are ubiquitous, so as with any organic material in nature, a wide variety of microorganisms is associated with the material collected by attines. Different bacteria, yeasts, and filamentous fungi have been recorded from attine fungus gardens. Leafcutters, for instance, take a wide diversity of endophytic fungi (**Table 1**) (and presumably other microorganisms) into their nests within cut leaf fragments (Rocha et al., 2014) and there is every reason to suspect a similar influx of microorganisms with foraging across the *Attina*. For millions of years, then, the attines have been introducing these microorganisms to their fungus gardens. By becoming fungus farmers, they established a complex symbiotic network of microorganisms within their colonies. Some of these microorganisms can be beneficial. The filamentous bacterium *Pseudonocardia* (*Actinobacteria*), for instance, is involved in an obligatory tripartite mutualism with some attine ants (especially leafcutters of the genus *Acromyrmex* but also lower attines), providing fungicidal or fungistatic substances that the ants use to protect their fungus gardens and themselves (Currie et al., 1999b; Mattoso et al., 2012; Li et al., 2018). On the other hand, facultative interactions have been shown with other bacteria such as *Burkholderia* (*Betaproteobacteria*) (Santos et al., 2004; Francoeur et al., 2021) *Streptomyces* (Kost et al., 2007; Meirelles et al., 2014) and other *Actinobacteria* genera (Sen et al., 2009). Meanwhile, other members of the community present in fungus gardens may have what seems to be a more negative interaction with the ant-fungus mutualism, as is the case with *Trichoderma* (*Ascomycota*: *Hypocreales*; Rocha et al., 2014), *Syncephalastrum* (Barcoto et al., 2017; Bautz et al., 2023), black yeasts (*Ascomycota*: *Phialophora*; Little and Currie, 2008) and the *Escovopsis* group. What emerges from these studies is the understanding that there may be many organisms acting in many different ways within an attine fungus garden and although some symbiotic relationships have already been studied, the vast majority of these relationships are still unclear (**Figure 2**).

Considering that the attine – mutualistic fungus relationship is the heart of the attines’ colonies, the knowledge of a parasite’s ability to affect this relationship is vital to understand the evolution and ecological success of these insects. The genus *Escovopsis* has been considered as the only specialised and highly virulent parasite of the mutualistic fungus, this notion even finding its way into undergraduate textbooks (Stearns and Hoekstra, 2005; Begon et al., 2006) and popular science texts (Wilson and Hölldobler, 2009) – see section 5 below. Thus, the interaction between the mutualistic fungus/ant symbiosis and *Escovopsis* is probably one of the most investigated relationships found in this system. However, in the light of broader studies it seems there was an oversimplification of the biology and ecology of this group of fungi, so the hypothesis that *Escovopsis* is a highly virulent parasite deserves further examination.

## 3 The genus *Escovopsis* and relatives

### 3.1 Taxonomic history of *Escovopsis* and relatives

The taxonomic history of the genus *Escovopsis* up to the 21st century is given by Augustin et al. (2013) so we only present that briefly here. The story of *Escovopsis*, although it was yet to be named such, began with Möller (1893), who considered it to be an asexual state of the mutualistic basidiomycete fungus of the attines. Subsequently, other scientists produced illustrations of this mysterious fungus. Stahel and Geijskes (1941) and Weber (1966) observed it growing in nests of fungus-growing ants, the latter correlating its presence with “abnormal circumstances” in a colony of *Trachymyrmex septentrionalis*. Afterwards, this same fungus was identified and described by Kreisel (1972) as *Phialocladus zsoltii*, using an isolate associated with *Atta insularis* (*Hymenoptera*: *Formicinae*: *Attini*), in Cuba. Since Kreisel had not determined a holotype for *Phialocladus* at the time, the name was considered invalid and the genus was re-described by Muchovej and Della Lucia (1990) as *Escovopsis*, in reference to the brush-like vesicles formed on the conidiophores (**Table 1**; Note: Brush in Latin is *penicillo*, a term already used for a well-known fungal genus with brush-like conidiophores. In modern Portuguese, however, brush is *escova*, whose etymological origin is the Latin *scopa*. The original description of *Escovopsis* considered the Portuguese word *escova*). A second species, *E. aspergilloides*, was described shortly after (Seifert et al., 1995), being distinguishable principally due to its globose *Aspergillus*-like vesicles. It is indeed the presence of conidiophores with vesicles that is the most remarkable morphological feature of *Escovopsis* (treated in full by Montoya et al., 2021).

In the nearly three-decade interval up to 2013, with only these two species, *E. weberi* and *E. aspergilloides*, described (Muchovej and Della Lucia, 1990; Seifert et al., 1995), it was common for different morphotypes to be described by their colony colouration (for example Gerardo et al. 2006; Masiulionis et al., 2015) (but see Montoya et al., 2021 for a more recent treatment). Studies focused predominantly on the possible parasitic nature of these fungi, addressing more ecological or evolutionary aspects rather than taxonomy and diversity (discussed in Montoya et al., 2021). Thus, fungi that are morphologically, physiologically and phylogenetically distinct were labelled *Escovopsis* because they share the same environment, yet without a proper examination of their morphology and phylogenetic placement for taxonomic purposes (Montoya et al., 2019). This changed from 2013 with the descriptions of three new species of *Escovopsis* and the erection of a new genus, *Escovopsioides*, which is phylogenetically related yet distinct from *Escovopsis* and also differs by an absence of pigmentation, lageniform phialides produced on terminal and intercalary, globose vesicles and by smooth conidia in long chains (Augustin et al., 2013).

Remaining with *Escovopsis*, two of the newly described species had morphological similarities with *E. weberi*, one having larger and more ornate conidia (*E. moelleri*), the other having smaller conidia (*E. microspora*). The third species was similar to *E. aspergilloides* in having globose vesicles yet could be characterised by its more ornamented conidia and slower growth in culture (*E. lentecrescens*) (Augustin et al., 2013). All of these were found in fungus gardens of two subspecies of *Acromyrmex subterraneus*, from the same fragment of Atlantic forest in southeast Brazil. Another five new species, *E. atlas*, *E. pseudoweberi*, *E. catenulata*, *E. primorosea*, and *E. longivesica*, were described from Argentina, also isolated from nests of *Acromyrmex* ants (Marfetán et al., 2018). These species were described based on morphological differences of the vesicles and on shape and colour of the colonies. Phylogenetic data for these five new species were also provided but only for a few molecular markers (Marfetán et al., 2018). Two more species (*E. clavatus* - an orthographic variant of *E. clavata* - and *E. multiformis*) were described from southern Brazil, from the basal attine *Apterostigma*, and these form a third group within the *Escovopsis* clade, both phylogenetically and also morphologically (Montoya et al., 2019). Both species possess a curious swelling in the mid-region of the terminal conidiophore but their vesicles are also distinct, being either clavate (*E. clavata*) or variable (globose, subglobose to clavate in *E. multiformis*) (Montoya et al., 2019). While it has been proposed that globose vesicles represent the ancestral state when compared with cylindrical vesicles (Meirelles et al., 2015a), this newer group, especially the possession of both forms by *E. multiformis*, has cast doubt on this. In 2023, after assessment of a collection of more than 350 *Escovopsis* isolates, another 13 *Escovopsis* species were introduced in the genus, based on standardised criteria of culture media to grow cultures as well as diagnostic morphological and phylogenetic characters (Montoya et al., 2023). In this taxonomic treatment, *E. microspora* was considered to be a morphological variant of *E. weberi* and was synonymised as such. In MycoBank (a nomenclatural repository of fungal names; https://www.mycobank.org/) there are a total of 24 accepted *Escovopsis* species names to date, but it is likely that more species, with new morphological conformations, will be described in the near future.

Until recently, there was a lack of standardisation in the description of new species within this group. This was first addressed by Montoya et al. (2019) in an effort to expand the range of growth media and conditions (especially temperature) in which new species’ characteristics could be described. In subsequent studies, Montoya et al. (2021, 2023). proposed standardised conditions, including the addition of two new molecular markers and detailed macro- and microscopic morphological evaluations. These authors used these criteria to reassess the genus, hopefully setting the standard henceforth for descriptions of new species.

In the intervening period, two new species considered at the time to be *Escovopsis* had been described from lower attines in southern Brazil (*E. kreiselii*; Meirelles et al., 2015b) and southeast Brazil (*E. trichodermoides*; Masiulionis et al., 2015). The subsequent reconsideration of the genus *Escovopsis* indicated that these two species did not belong to this genus. They were therefore assigned to two new genera and renamed *Sympodiorosea kreiselii* and *Luteomyces trichodermoides*, respectively (Montoya et al., 2021). Both of these remain, at the time of writing, genera comprised of single species, but more species will likely be described in the future, especially in *Sympodiorosea*.

The three genera to have emerged from this exercise, *Escovopsioides*, *Sympodiorosea* and *Luteomyces*, are sister genera related to *Escovopsis* (Augustin et al., 2013; Montoya et al., 2021) (**Figure 1**). While they all belong to the family Hypocreaceae, they form separate monophyletic clades, *Luteomyces* being the group closest to *Escovopsis* and *Sympodiorosea* closest to *Escovopsioides* (Montoya et al., 2021). In addition to phylogenetic division, these species also have morphological peculiarities that place them in distinct genera. Although *Escovopsioides* produces phialides on vesicles much as *Escovopsis* does, it is distinct in that it presents lageniform (flask-shaped) phialides arranged in terminal and intercalary vesicles, in addition to differences in the form of the conidia. *Sympodiorosea* has sympodial (side-branching) conidiogenous cells as the main characteristic of the genus and also has pink-coloured colonies, while *Luteomyces* presents conidiophores with synchronous conidiogenous cells and yellow-coloured colonies. It is salient to point out that a number of studies to date have described what were considered to be *Escovopsis* isolates as “brown”, “pink” or “yellow”, so it is likely that some may actually belong to these new genera (e.g. Gerardo et al., 2006a, b). Intriguingly, the above morphological characteristics are not observed in any other genus of the Hypocreaceae, so it may be that they have arisen as a result of selection related to the particular life styles of these fungi in association with the ants.

These recent findings open interesting possibilities for the study of this system. The formal description of new genera expands the known diversity of fungi that associate with the attine system and can exploit it. Future studies may reveal how these different fungi may interact differently with attine ant nests and potentially even with one another, if they are found to co-occur.

### 3.2 Macroevolution and biogeography

The geographical distribution of *Escovopsis* has been little explored. None of the four genera under consideration here have ever been found in the absence of an association with attine fungus gardens. Thus, it is expected that *Escovopsis* species are limited to the geographical distribution of fungus-growing attine ants: exclusively the Americas and mainly the tropics and subtropics (Mayhé-Nunes and Jaffé, 1998).

Since *Escovopsis* (plus *Escovopsioides*, *Sympodiorosea* and *Luteomyces*) has not been found outside the attine system, it has been hypothesised that it coevolved in a tripartite relationship with the attine ants and the mutualistic fungus since the beginning of fungal domestication (Currie et al., 2003; Mehdiabadi and Schultz, 2010). The scenario accepted so far is that this genus was probably a parasite of free-living leucocoprineous fungi and has followed the evolution of fungiculture practised by the attines since then (Currie et al., 2003; Gotting et al., 2022). This hypothesis is reasonable, given that the group belongs to the family *Hypocreaceae*, which contains other mycoparasitic fungi such as *Hypomyces*, *Cladobotryum* and *Trichoderma*. However, the order *Hypocreales* to which these fungi belong also contains members that are noteworthy as parasites of arthropods (e.g. *Cordyceps*, *Metarhizium*), parasites of plants (e.g. *Fusarium*) or as endophytes (*Epichloë*), with strong support for host-switching through the group’s evolutionary history (Spatafora et al., 2007; Vega et al., 2009). It is worth keeping an open mind, then, about the group’s evolutionary history and being aware of the possibility that some members may retain the capacity (or the molecular toolkit) to exploit other modes of life such as endophytism - see *Trichoderma* and *Metarhizium* as examples of this (Vega et al., 2009; Woo et al., 2023).

Within this scenario of these fungi coevolving with the attine-basidiomycete mutualism, it was initially proposed that a pattern of co-cladogenesis would be found, based principally on evidence from *Escovopsis* infecting *Trachymyrmex* nests in Central America (Currie et al., 2003). This view has been eroded subsequently, beginning with the finding of so-called “brown *Escovopsis*” infecting the pterulaceous ‘coral fungus’ of *Apterostigma* (Gerardo et al., 2006b) (and also reviewed in Mehdiabadi and Schultz, 2010), rather than being restricted to higher attines as had been expected; while these authors maintained the co-cladogenesis model, they did provide evidence for ‘occasional’ switches of *Escovopsis* or its relatives between lineages of the host ants. Further evidence of frequent host switching was subsequently found in *Escovopsis* associated with the more derived leafcutter genera *Atta* and *Acromyrmex* (Taerum et al., 2007). On the flip side, subsequent studies showed that individual nests can host multiple strains of *Escovopsis* (Taerum et al., 2010) and then that multiple species of *Escovopsis* (and also the new genus *Escovopsioides*) can be found in nests of a single species of *Acromyrmex* (albeit with two subspecies) in a single forest fragment in southeast Brazil (Augustin et al., 2013). The strongest evidence against co-cladogenesis, though, came from a study using isolates from across Central and South America (but still, as the authors point out, with limitations in the representativeness of the samples (Meirelles et al., 2015a). These authors clearly showed that – in the higher attines at least – there is no overall pattern of fidelity or co-cladogenesis of *Escovopsis* species to their ant-fungus hosts. This does not, however, mean that there may have not been more isolated cases of such, and this may even be expected where there are geographical barriers, as in the Caribbean islands or the Andes etc. It also does not mean that there may not have been co-cladogenesis at the higher taxonomic level, i.e. with *Sympodiorosea* and *Luteomyces* in particular, given these have only been found in lower attines to date.

With the exception of *E. aspergilloides* described from Trinidad, all described species within the *Escovopsis* group have been isolated in southern South America (Minas Gerais, São Paulo and Santa Catarina states in Brazil, or in Argentina). Isolates from other regions that have been the objects of evolutionary or ecological studies bear many similarities with these, most obviously colony pigmentation and association with ant genera. Nevertheless, until these two sets of studies are fully integrated, our picture of the group’s evolutionary history and its biogeography remains sketchy.

Meirelles et al. (2015a) suggested that *Escovopsis* species could present a latitudinal diversity gradient, in which there is a reduction of diversity at higher latitudes. However, a greater sampling effort is needed to test this hypothesis, in addition to including larger samples from basal attines species (like *Apterostigma*). This also applies to *Escovopsioides*, *Sympodiorosea* and *Luteomyces*, the more recently described genera found in fungus gardens.

### 3.3 Biological cycle

It should be evident that to understand any parasite it is necessary to have some understanding of its life cycle, not just to have a handle on its ecology but also to understand what selective forces may be at play. Some basic questions are what hosts can be infected and how the parasite is transmitted between hosts. Regarding the first of these questions, *Escovopsis* has only ever been isolated from attine fungus gardens and their waste dumps (Augustin et al., 2017), so it seems reasonable to suppose that attine nests are their only habitat until it is found elsewhere. Given the evolutionary history of the *Hypocreales*, as mentioned above, it is worth considering the possibility that these fungi can also infect plants as endophytes (see Pereira et al., 2024). They have yet to be isolated from plants, however. If these fungi did infect plants, they might be found in leaf material being carried by leaf cutters. However, where such material has been examined, being transported by two species of *Atta*, these fungi were not found while many others, including *Trichoderma* (which is in the same family as *Escovopsis*), were (Rocha et al., 2014, 2017).

How *Escovopsis* is transmitted between colonies is unknown. It has not been detected in newly founded colonies (Currie et al., 1999a) or in the fungal pellets carried by the alate gynes (Moreira et al., 2015), so horizontal transmission has always been considered to be the main and most likely way in which the fungus can reach new colonies and complete its life cycle (Currie et al., 1999a). It is important to note, however, that transmission between colonies has never actually been observed, even in laboratory conditions. We are aware of only one study that has addressed this explicitly, by assessing the waste being discarded from *Acromyrmex* colonies (Augustin et al., 2017). Here, *Escovopsis* was found in every waste dump sampled (111 waste samples from 34 *Acromyrmex* colonies) in a small area of an Atlantic forest fragment and in a few of these cases, it was actively sporulating in the dump (Augustin et al., 2017). This indicates that it may leave nests – which would be the first step in horizontal transmission – when taken out with waste material. It is worth noting, however, that many attines dump their waste underground, so *Escovopsis* leaving nests via this route seems improbable in these cases. Another possibility is that *Escovopsis* leaves nests when the nest dies and it can be found sporulating on dying fungus gardens that have become accessible (to other invertebrates for example) due to the lack of ants (Hart, 2002).

Even if *Escovopsis* emerges from a nest, it must still reach other nests, specifically the underground fungus garden, begging the question of how it might do this. Some species of *Escovopsis* have ornamented conidia, suggesting that phoresy is a possibility. The potential for *Acromyrmex* to carry spores of *E. moelleri* has been shown empirically (Augustin et al., 2017), but this is still several steps from infecting a new colony, especially as ants are perhaps one of the arthropods least likely to enter another ant nest. Ant nests do host many other arthropods, however (Sumner et al., 2003; Campbell and Crist, 2016; Phillips et al., 2021), including social parasites and mites, both of which could easily carry spores phoretically and may actively seek to enter other ant nests, perhaps of different species from their original hosts. This would effectively be vector-borne transmission between nests and seems quite feasible. The possibility that spore morphology may confer an ability to be phoretic on arthropods has been raised for *E. moelleri*, with preliminary tests suggesting that it may indeed be a possible means of horizontal transmission between colonies (Augustin et al., 2017).

Finally, it is worth mentioning airborne and waterborne transmission as possibilities. It is a tenet of microbiology, after all, that “Everything is everywhere, but the environment selects.” (Becking, 1934). Again, though, either possibility would require investigation and it is worth noting that to date no members of this group have been found with characteristics typical of fungi that disperse with the aid of water, such as a mucilaginous layer on spores for example (De Menezes et al., 2015).

The above modes of transmission between nests are (or would be) all horizontal. Vertical transmission between nests was discarded in the literature quite early on, based on one study with *Atta colombica* (Currie et al., 1999a). In that study, the fungal pellets carried by virgin alates in their infrabuccal pockets were screened for *Escovopsis* using culture-dependent methods. None of the 38 fungal pellets analysed were positive for *Escovopsis*. As alluded to above, this has led to statements common in the literature that this fungus is transmitted horizontally, with no actual evidence for the latter. This view has even found its way into academic textbooks and popular science books (Stearns and Hoekstra, 2005; Begon et al., 2006; Wilson and Hölldobler, 2009). As we argue below (section 6), further investigation of the possibility of vertical transmission is evidently needed before it can be discarded.

To review the possibility of vertical transmission, we must first review how colonies are founded and how the basidiomycete mutualist is transmitted vertically between colonies. Before her nuptial flight, the reproductive alate female gathers fragments of the fungus garden and stores them as a pellet in the infrabuccal cavity (von Ihering, 1898; Huber, 1905). After mating, the future queens fall to the ground and dig a chamber where the mutualistic fungus is regurgitated and cultivated using faecal material and eggs, until the first workers can emerge and start foraging (Augustin et al., 2011). Fungal pellets from *Atta* spp., as well as gardens of incipient laboratory colonies and cuticles of foundress *Atta* queens, have been sampled and cultured using culture-dependent methods, but *Escovopsis* has never been detected (Currie et al., 1999a; Pagnocca et al., 2008; Moreira et al., 2015). The earliest detection of *Escovopsis* in an attine colony coincided with the moment when the first workers started foraging, suggesting that the fungus arrives from an external source (Moreira et al., 2015).

*Escovopsis* is found in nests of *Atta*, *Acromyrmex*, *Trachymyrmex*, *Sericomyrmex* and *Apterostigma*. Examination of infrabuccal pellets, however, has only been done with *Atta* species (Currie et al., 1999a; Pagnocca et al., 2008; Moreira et al., 2015; Authors pers. obs.). The nuptial flight in *Atta* spp. is a phenomenon that is hard to miss, so it is relatively easy to collect gynes, wait for them to regurgitate their infrabuccal pellets and examine these. This is not the case, however, in other attine genera such as *Sericomyrmex* and *Apterostigma*. It is possible that vertical transmission occurs in attine genera that have yet to be examined and it is worth striking a note of caution about the whole genus *Escovopsis* and its relatives based on studies from only one genus of ant host. Thus, future studies should assess the infrabuccal pellets of the other ants with which *Escovopsis* is associated to draw a general conclusion regarding the life cycle of this group of fungi.

As mentioned above, all of the studies aimed at assessing the possibility of vertical transmission of *Escovopsis* have relied on culture-dependent methods. It is likely that one or more of the other fungi present in the pellet, which are saprotrophic fungi, air contaminants, soil-borne fungi, endophytes, or other mycoparasites (Rodrigues et al., 2005b, 2008), can prevent *Escovopsis* growth on culture media. It is also possible that at least some *Escovopsis* species can be vertically transmitted if dormant spores are taken into the infrabuccal pellet. In this scenario, *Escovopsis* could strategically remain dormant at the beginning of the development of the colonies until a certain amount of time has passed or it finds suitable conditions to grow. If so, they would not be detectable by culture-dependent methods in infrabuccal pellets or in very young fungus gardens. Dormancy has been observed in spores of *E. moelleri* (Augustin et al., 2017), supporting this possibility. However, the same fact can also invalidate the vertical transmission hypothesis, because if dormancy is broken by the presence of the host, then why was it never detected from the moment that the queen starts growing the fungus garden? There are several possible answers to this question: First, the queen may release substances that inhibit the growth of *Escovopsis*. Some ants, such as *Acromyrmex octospinosus*, have certain cleaning behaviours such as autogrooming and the addition of faecal liquids to the plant substrate incorporated into the fungus garden, thereby preventing the growth of parasites, and the young queen may employ such strategies (Fernández-Marín et al., 2003). Furthermore, queens also use metapleural gland secretions as a prophylactic measure against pathogenic bacteria and fungi (Hölldobler and Wilson, 1990). Therefore, these defences might be preventing the initial growth of *Escovopsis*. Finally, we cannot discard the possibility that the dormancy-breaking mechanism involves processes and conditions that are much more complex and specific than just the presence of the mutualistic fungus of the attine ants. An endogenous mechanism that delays germination until the colony is more established could mean that *Escovopsis* is only found (coincidentally) once the ants begin foraging outside the colony.

Undoubtedly, knowing more of the life cycle of *Escovopsis* will help researchers to understand the ecology and evolution of the genus and its relationship with the fungal cultivar and the rest of the colony. It could be interesting to invest in the creation of specific primers to detect *Escovopsis* by culture-independent methods. In this way, it could be possible to investigate how the fungus reaches the colonies and whether it is vertically or horizontally transmitted, by sampling the pellets carried by the queens, the fungus garden of incipient colonies (in different conditions of light, temperature and humidity) and the material collected by the ants (to feed their cultivar). An interesting case is an invasive attine, *Acromyrmex octospinus*, which was apparently accompanied by *Escovopsis* in its arrival on the island of Guadeloupe, raising questions about the possibility of vertical transmission with dispersing gynes (Meirelles et al., 2015a).

## 4 *Escovopsis* as a mycoparasite

Fungicolous fungi are consistently found in association with other fungi and may have a range of interactions with their host that includes mycoparasitism (Barnett, 1963; Rudakov, 1978; Jeffries, 1995; Sun et al., 2019). Indeed, most fungicolous fungi are mycoparasites (Sun et al., 2019). While our focus here is on mycoparasites within the *Ascomycota*, this lifestyle is found in diverse phyla such as *Basidiomycota, Blastocladiomycota, Chytridiomycota, Entomophthoromycota, Kickxellomycota, Mucoromycota* and *Rozellomycota* (reviewed by Sun et al., 2019).

Mycoparasites are mostly categorised by the manner in which they acquire nutrients from their hosts, being divided into biotrophs and necrotrophs (Jeffries, 1995), although this can be considered a continuum (see Sun et al., 2019). Biotrophic mycoparasites have an obligatory relationship with their hosts and usually a narrow host range, using living cytoplasm as their source of nutrition while causing limited damage (Barnett and Binder, 1973). They penetrate the tissue of their hosts through specialised hyphae, then obtaining nutrients released by the host (Deacon and Berry, 1992; Jeffries and Young, 1994; **Table 2**). They usually have slower growth and are less competitive than necrotrophs (Deacon and Berry, 1992). Meanwhile, necrotrophic fungi kill their hosts (at least locally, considering that fungi are modular organisms), using specialised structures in addition to secreted enzymes and antifungal compounds, with subsequent use of the necromass as a source of nutrients (Deacon and Berry, 1992; **Table 2**). These fungi generally have a comparatively broad host range (Jeffries and Young, 1994; Borkovich et al., 2010; **Table 2**).

**Table 2:** Characteristics of other mycoparasites compared to *Escovopsis* and relatives.

Characteristics	Examples	Has this been observed in any *Escovopsis* species?
Biotrophic mycoparasites. Parasitic fungi that obtain nutrients from live mycelium of the host (Barnett, 1963).	*Ampelomyces quisqualis* (Kiss et al., 2004), parasitic on powdery mildews and the basis for many biocontrol products. Among the first mycoparasites described. Biotrophic in the earlier stages of the interaction, becoming more necrotrophic.	Yes – shown in *E. weberi* (Marfetán et al., 2015). This species was considered a biotrophic mycoparasite because of the penetration of the host hyphae from the presence of structures such as hooks (Boosalis, 1964)
Necrotrophic mycoparasites. Destructive parasitic fungi that kill their host to obtain nutrients (Barnett, 1963).	*Clonostachys* spp., *Trichoderma* spp.	Yes – shown in *E. weberi* (Reynolds and Currie, 2004)
Wide range of hosts	*Clonostachys rosea*, *Trichoderma viride* (Gams et al., 2004; Mukherjee et al., 2013).	No, limited to the basidiomycetes that live in symbiosis with attine ants.
Host-specificity at the genus or family level	*Hypomyces* that parasitise agarics (Rogerson and Samuels, 1989; Tamm and Põldmaa, 2013).	Yes (Currie et al., 2003)
Formation of appressoria-like infection structures or hyphal swellings at the points of interaction with host	*Trichoderma* spp (Chet et al., 1981; Lu et al., 2004)	No
Specialised structures to penetrate the host. Typical of invasive necrotrophic fungi (Jeffries, 1995).	*Trichoderma* spp (Chet et al., 1981)	Yes – shown in *E. weberi* (Marfetán et al., 2015)
Coiling of parasite hyphae on host hyphae. Typical of contact necrotrophic fungi (Jeffries, 1995).	A*rthrobotrys oligospora* (Olsson and Persson, 1994; Singh et al., 2012), *C. rosea* (Abdellatif et al., 2022), *Trichoderma* spp (Elad et al., 1983; Lu et al., 2004)	No. Although similar coiling contacts were observed for *Escovopsis* sp. (Varanda-Haifig et al., 2017) and *Sympodiorosea* (*Escovopsis*) *kreiselli* (Custodio and Rodrigues, 2019)
Production of anti-fungal chemicals during parasitism. Typical of non-contact necrotrophic fungi (Jeffries, 1995).	*Clonostachys* spp (Karlsson et al., 2015)	Yes – shown in *E. weberi* (Reynolds and Currie, 2004) and *Escovopsioides nivea* (Varanda-Haifig et al., 2017)
Chitinases present. Enzymes important for degradation of cell wall of host fungus during mycoparasitism.	*Trichoderma reesei* (Kubicek et al., 2011); *T. harzianum* (Zeilinger et al., 1999); *T. atroviride* (Reithner et al., 2005)	Yes – shown in *E. weberi* (De Man et al., 2016)
Volatile compounds.	*Trichoderma atroviride* (Stoppacher et al., 2010)	Yes* (Masiulionis and Pagnocca, 2020)
Nutrient transfer from host fungus.	*Arthrobotrys oligospora* (Olsson and Persson, 1994)	No

*The *Escovopsis* isolates used in this study were not identified.

*Escovopsis* is presumed to have coevolved with the attines and their symbiotic fungus (Currie et al., 2003; Gotting et al., 2022) and the first studies of interactions between it and the mutualistic fungus assumed a tight association between the groups: *Escovopsis* clades were specifically associated with certain ant clades (Currie et al., 2003; Mehdiabadi and Schultz, 2010). However, it was known that different higher attines can share the same *Escovopsis* (Taerum et al., 2007; Meirelles et al., 2015a). Meanwhile a single fungus garden may host multiple *Escovopsis* strains (Taerum et al., 2010; Augustin et al., 2013; Christopher et al., 2021).

Specialised structures have been observed in some *Escovopsis* isolates, but considering the vast diversity of undescribed species, it is clear from Marfetán et al. (2015) and Varanda-Haifig et al. (2017) that additional studies are needed to determine the variation in strategies of exploitation of the host fungus. There is a diversity of mechanisms for mycoparasitism employed within the genus *Trichoderma* (Atanasova et al., 2013; Mukherjee et al., 2022; **Table 2**), so it is likely that increased research effort on *Escovopsis* and relatives may reveal a diversity of mechanisms within this group or that species may have lifestyles other than parasitism.

Chemical interactions of *Escovopsis* with its host have been better studied. *Escovopsis* species produce chemical compounds that inhibit the growth of the fungus grown by ants (Varanda-Haifig et al., 2017). In addition, these compounds can inhibit bacteria mutualistic with the ants (Boya et al., 2017; Dhodary et al., 2018; Heine et al., 2018) and even harm the ant workers (Heine et al., 2018). However, there are no reports so far that *Escovopsis* influences the growth of fungi other than the ants’ mutualist and other *Escovopsis* isolates, the latter probably through the production of secondary metabolites (Christopher et al., 2021). The degree of specialisation of *E. weberi* to its host is reflected in its reduced genome compared to mycoparasitic relatives within the *Hypocreales*. Genome sequencing has shown that it has lost genes related to carbohydrate-active enzymes (De Man et al., 2016). While the obvious conclusion is that it relies on its host for this aspect of its nutrition, this could just as easily be what is left over after the host has degraded its substrate, rather than *Escovopsis* taking carbohydrates directly from the host. Meanwhile, it upregulates genes during attack that are responsible for degradation of host cell walls (De Man et al., 2016).

Whether volatile organic compounds produced by *Escovopsis* affect host fungi such as *Leucoagaricus gongylophorus* is difficult to assess due to the latter’s exceedingly slow growth *in vitro*. Nevertheless, the identities of these volatiles have led to the suggestion that they could be harmful to both *L. gongylophorus* and its ant partners (Masiulionis and Pagnocca, 2020). Meanwhile, volatiles produced by *L. gongylophorus* can accelerate the growth of *Escovopsis*, so chemotropism has been suggested (Masiulionis and Pagnocca, 2020). It has further been suggested that the volatile organic compounds (VOCs) produced by *L. gongylophorus* maximise *Escovopsis* growth, potentially helping to explain its rapid growth in the presence of its host (de Oliveira et al., 2024). This hypothesis was based on the parallel with VOC vitamins produced by soil microorganisms that can be used by other microorganisms as a nutritional source (Stotzky and Schenck, 1976). We stress that this would be extremely important given there are no studies, so far as we know, that prove the nutrient transfer from host fungus to parasite.

Further evidence of the specificity of the host-parasite interaction was shown in experimental assays of the effects of volatiles released by *L. gongylophorus* on spore germination in three species of *Escovopsis* (*E. weberi*, *E. lentecrescens* and *E. moelleri*). In all three cases, exposure to *L. gongylophorus* volatiles markedly increased germination, while exposure to volatiles from another basidiomycete appeared to inhibit this (Augustin et al., 2017).

## 5 *Escovopsis* as a parasite of attine colonies

### 5.1 Is *Escovopsis* virulent at the colony level?

*Escovopsis* is a common inhabitant of attine ant gardens, with estimates of prevalence varying from 18 to 75%, depending on the ant species and location (Currie, 2001; Gerardo et al., 2004; Rodrigues et al., 2005a, 2008; Augustin et al., 2013; Reis et al., 2015; Pereira et al., 2016). These figures certainly indicate that there may be many situations in which attine colonies can persist while harbouring this symbiont (or at least the species and strains found in these studies). Laboratory colonies can appear to be perfectly healthy while harbouring *Escovopsis* – in fact, it is extremely difficult (at least within the native range of these organisms) to ensure the absence of *Escovopsis* from colonies being studied (Authors Pers. Obs.). *Escovopsis* can sometimes be found in colonies in decline (Currie et al., 1999a; Hart, 2002) but this of course does not indicate the fungus is responsible for the state of the colony. There are prominent examples in the host-parasite literature of secondary infections being overly apparent in declining hosts, as with microsporidioses in HIV-immunocompromised humans (Didier and Weiss, 2006) or of opportunistic organisms exploiting dying or dead hosts, as with insects killed by *Bacillus thuringiensis* or *Metarhizium* spp. yet colonised by gut bacteria (Raymond and Bonsall, 2013; Wang et al., 2023).

Why then are unfounded claims for *Escovopsis*, such as it being a “particularly devastating enemy of the fungus”, “virulent”, “horizontally transmitted” and “highly virulent, able to devastate ant gardens and thus doom the entire colony” to be found in textbooks (Stearns and Hoekstra, 2005; Begon et al., 2006) and popular science books (Wilson and Hölldobler, 2009)? To understand this, we trace here the history of *Escovopsis* being described as a virulent parasite. This fungus came to be of particular interest after a seminal study by Currie et al. (1999a) and that study warrants particular attention. In it, the frequent isolation of *Escovopsis* (sensu lato - s.l. - includes in this definition *Escovopsis* and all relative genera not known at the time) from attine ant colonies (26% of all contaminants found in more than 2,400 garden pieces) and the verification of Koch’s postulates, led *Escovopsis* to be considered a specialised parasite. Although Koch’s postulates were applied, the age and size of ant colonies are likely to shape the outcome of an infection – small and young colonies may be more vulnerable to *Escovopsis* infections, with greater negative fitness effects of these. Koch’s postulates are important steps to indicate the causal agent of a disease, but Robert Koch himself recognised the limitation of his approach. These barriers were not discussed or questioned in the original text that suggested the pathogenicity of *Escovopsis* through the postulates (Currie et al., 1999a). Perhaps the most problematic issue is the fact that we are not dealing with an individual, but rather a eusocial organism and its symbiont. Although *Escovopsis* is considered a specialised mycoparasite of the mutualistic fungus of the attine ants, the effect caused by it in certain situations (especially in those where the colony is already suffering a disorder – de Mendonça et al., 2021) affects the entire system. Besides, it is very common to isolate *Escovopsis* from healthy colonies that are normally foraging, both in the field and in the laboratory (Currie et al., 1999a; Gerardo et al., 2004; Rodrigues et al., 2005a; Augustin et al., 2013). Consequently, it is rarely possible to identify if a nest is infected by *Escovopsis* – this can only be determined when it is being overgrown or by isolating the fungus – there are no ‘symptoms’ beyond the presence of the fungus that can be attributed to a ‘disease’ caused by the fungus. Meanwhile, it is impossible to verify whether a colony is free of *Escovopsis* by culture-dependent methods, as total sampling of a fungus garden would require its destruction. Perhaps, in the future, a sampling plan of fungus gardens could be devised based on extensive sampling, that might allow one to determine the probable infection status of a fungus garden or colony. This would be a major effort but of tremendous use for guiding future studies.

During this same study under consideration (Currie et al., 1999a), young colonies of *Atta colombica*, between 6 and 8 weeks old, with fungus gardens of 60 to 75 ml were used. Such incipient colonies are fragile and do not have the same defence capability as mature colonies. The impact of *Escovopsis* infection in this study could well be ascribed to this fact. Also, *Trichoderma*, a well-known necrotrophic mycoparasite fungus, was used as a positive control for high inoculation of a proven aggressive fungus. However, the authors reported that they were unable to recover either it or *Escovopsis* at the end of the experiment. Two further issues require addressing: firstly, the authors did not mention whether they tested the viability of the conidia of both fungi. This test is common and essential in infection experiments to confirm if the conidia are capable of infecting the host. Therefore, it is possible that *Trichoderma* conidia were not able to infect colonies in this study (this has been shown elsewhere for *Trichoderma* – Rocha et al., 2017). Secondly, it is not possible to know whether the *Escovopsis* recovered from fungus gardens at the end of the experiment is the same as that which was inoculated in the nests. Even though the colonies had been labelled as *Escovopsis*-free, as explained above, it is not possible to state this by the methods used. In the face of everything that we have discussed here, we consider that evaluating the loss in colony fitness due to the parasite under these conditions is not the most appropriate method, especially because it is a complex system that involves different symbiotic associations (see **Figure 2**). The authors also stated that they had demonstrated horizontal transmission in this system, which they had not, and that high virulence would be consistent with virulence evolution under this mode of transmission. This latter point was not necessarily true at the time (Ewald, 1994; Frank, 1996; Dieckmann et al., 2002) and this remains the case now (Alizon et al., 2009; Cressler et al., 2016).

How then can we assess the virulence of *Escovopsis*? The broadest definition of virulence is the *harm* a parasite does to its host, while the definition of most relevance in terms of evolutionary ecology is the negative effect on the host’s *fitness*. It is the production of alate reproductive females bearing the fungal mutualist in their infrabuccal cavity and their capacity to found new colonies where the fitness of the pair of mutualists is actually expressed. (Note that this is an obligatory mutualism so both partners must be present in new colonies for fitness to be positive). Thus, even if *Escovopsis* takes nutrients directly from the symbiont as a biotroph or kills its hyphae as a necrotroph, if this interaction does not lead to a net decrease in the number of alate reproductives (and their founding of new colonies etc) then it is not actually a parasite (**Table 1**).

Unfortunately, we are unlikely ever to be able to design experiments in which we can assess the effects of any symbiont of the larger attine colonies on their production of reproductives (mature *Atta* colonies can be compared in size and function to adult elephants, so the prospect of replicated laboratory experiments is distant). Measuring fitness itself can be difficult, even for animals that have a solitary existence. It is therefore common in the study of animal parasites to assess life history traits as proxies for fitness (e.g. Elliot et al., 2005). Perhaps the most fundamental of these are survival and growth and, if feasible, the otherwise observed relationships of these proxies to eventual fitness. In the case of a social insect with a fungal symbiont, we could assess survival or population size of the insects, or quantity of the symbiont. We could also assess activity such as foraging. Negative effects on any of these might indicate (as proxies) negative effects on fitness and can also be considered “harm” in the broad definition of virulence. After Currie’s studies (Currie et al., 1999a; Currie, 2001), three studies have looked at this question with this type of approach (de Mendonça et al., 2021; Jiménez-Gómez et al., 2021; Queiroz et al., 2024) – see below.

The apparent absence of vertical transmission in *Escovopsis* s.l. has been interpreted in the light of the theory on the evolution of virulence, to explain the apparently high virulence of this fungus (Currie et al., 1999a). In a general and simplified way, horizontally transmitted parasites may be more virulent than vertically transmitted parasites as the latter rely on their hosts for transmission (Ewald, 1994). Using this to conclude that horizontally-transmitted parasites are highly virulent is, clearly, logically flawed. In fact, it is difficult to apply the extant theory to a given system, especially one as complex as those under consideration. Here, the hosts are superorganisms rather than individuals, they can live for years and they have a plethora of microbial symbionts. Meanwhile, we have only basic knowledge of some important details of the parasites, such as their mode of transmission, prevalence, frequencies of multiple infections, duration of infections and damage caused to the hosts. On the other hand, the idea that *Escovopsis* can be characterised as highly virulent was based on the observation of a few strains (probably from the *E. weberi* clade). However, recent studies considering several species across the phylogeny of *Escovopsis* and different levels of complexity of the ant’s colonies concluded that the genus has an opportunistic nature (Jiménez-Gómez et al., 2021) and low virulence (de Mendonça et al., 2021). This was the case for several species from the *Escovopsis* group and at extremely high levels of inoculum.

In the absence of studies showing demonstrable negative effects at the *colony level* then, it is parsimonious to consider that it is not actually a virulent parasite at that level, even if we show negative interactions with the host itself: apparent parasitism.

Given the above argument that *Escovopsis* is not actually a virulent parasite, we are still left with the possibility that it does exert a cost on its host(s). What are the costs of this? *Escovopsis* most certainly uses the mutualistic fungus for its sustenance, so that cost is a given. Meanwhile, workers invest time, energy and chemical compounds in defence. This is itself a cost and we know that costs of anti-parasite defences can be subtle (Moret and Schmid-Hempel, 2000; Elliot et al., 2005). It also indicates a history of selection for defence, which is in turn indicative of the existence of costs over evolutionary history. Strictly speaking, these costs must be weighed against any benefits of the interaction. No benefits have ever been demonstrated. However, it is perfectly possible that such exist (as with resident gut flora in animals that increase resistance to parasites, for example). Without any demonstrable benefits, then, our best understanding is that there is a cost, albeit a moderate one. In other words, while *Escovopsis* is not a virulent parasite, it remains a mild parasite.

### 5.2 How do the hosts defend themselves?

How then do the ants and their mutualist partners defend themselves against *Escovopsis*? Phylogenetic analyses indicate that *Escovopsis* coevolved with fungus-cultivating ants (Currie et al., 2003), so it is expected that defensive strategies of the attine ants and their mutualistic fungus against this parasite have been shaped by evolution. The social organisation of the ants (‘social immunity’; Elliot and Hart, 2010; Cremer et al., 2018), hygienic behaviour and association with the actinobacterium *Pseudonocardia* (Currie et al., 1999b) are strategies that contribute to *Escovopsis* control within nests. In addition, *in vitro* results have shown that the fungus cultivated by ants can itself inhibit the growth of parasites (Gerardo et al., 2006a; Van Bael et al., 2009a; Pietrobon et al., 2022). These features can contribute to the reduction of a parasite’s virulence in social insects, in general, as discussed by Hughes et al. (2008). Perhaps the evolutionary pressures have been shaping *Escovopsis* for a strategy in which it remains in the colonies causing minimal damage and waiting for the most propitious moment (e.g. the queen’s death for any other reason) to actually overgrow inside the nest in an aggressive way.

Within colonies of ants and other social insects, tasks can be divided between individuals with different morphologies (polyphenism or polymorphism) and ages (age polyethism) (Wilson, 1980; Hinze and Leuthold, 1999). Schmid-Hempel (1998) compared the separation of tasks by age to a conveyor belt model, where more valuable young workers are responsible for safer duties inside colonies, and as these workers get older, they start to perform tasks outside the nest that have higher risks. If they do not return to the centre of the nest they are less likely to bring pathogens in with them. This time schedule is very well studied in bees and it is known that it can be accelerated if the colony is under stress (Natsopoulou et al., 2016). In other words, some workers may have a reduced life expectancy and, therefore, begin to perform more risky tasks, depending on the stress factors that the colony is suffering from. Furthermore, in bees it appears that less virulent parasites influence host behaviour less, in terms of accelerating the change with age of the individual in the performance of nest activities, than more virulent parasites (Natsopoulou et al., 2016). Social networks can also be adjusted in response to the risk presented by parasites (Stroeymeyt et al., 2018). It would be interesting to investigate whether this occurs with colonies experimentally infected with *Escovopsis* and other fungi found in ant colonies of the Attini tribe. This response may give us evidence of the host-parasite fidelity, the parasite’s virulence and the stability of the interaction.

### 5.3 Variation in host exploitation strategies

In the above arguments, for convenience we have considered *Escovopsis* a single taxonomic entity, despite this not being the case. However, we can find different strains of *Escovopsis* hosted by the same ant species and even sharing the same colony (Taerum et al., 2010; Augustin et al., 2013). Different strategies (e.g. infection, transmission or virulence strategies) are important for the survival and persistence of members of each species. Therefore, we expect variation between species or isolates in their strategies to exploit their hosts and thereby their virulences (as shown by Christopher et al., 2021; Jiménez-Gómez et al., 2021; Queiroz et al., 2024). Generalisations have been made for the whole of the genus *Escovopsis* (and by implication the other three genera), assuming it to be a highly virulent parasite and disregarding factors such as colony condition.

For *Escovopsioides* and one of the two new genera, *Luteomyces*, we are almost entirely ignorant as to their roles in the symbioses of the Attini. Preliminary studies have shown that *Escovopsioides* is an antagonist of the basidiomycete mutualist, but it appears to be less virulent than *Escovopsis*, causing minor negative effects on colonies (Varanda-Haifig et al., 2017; de Mendonça et al., 2021; Pietrobon et al., 2022). The other two genera were erected only very recently and there is only one study that has evaluated the interaction between *E. kreiselii* (now *Sympodiorosea kreiselii* Montoya et al., 2021) and its host, the fungus garden of a lower attine, showing that *E. kreiselii* was able to inhibit the mutualistic fungus in dual culture assays (Custodio and Rodrigues, 2019). Similar, previous, studies showed often similar interactions with what we now know to be species of *Sympodiorosea* (e.g. Gerardo et al., 2006a, b), at that point largely referred to as “pink *Escovopsis*”.

Considering that we now know that what we thought was one genus (with one described species, *Escovopsis weberi*) is now actually four genera (with 24 species described to date from just one of these and a range of morphologies and growth patterns), it seems that we have more lacunae regarding the interactions of this group of fungi with the ant-fungus mutualism than actual knowledge. Additionally, there is considerable diversity within the attine ants and the basidiomycetes involved, the substrate brought into the nests, the sizes of these colonies and their ecological contexts. We suggest therefore that the virulence of the *Escovopsis* clade towards its hosts is far more complex than a simple description as highly virulent for all genera, especially given the importance of context-dependency in host-parasite interactions (e.g. Elliot et al., 2002; Mitchell et al., 2005). While simplifying our view of this system facilitates research and makes it possible to carry out numerous studies, it can be a long way from reality. We need to consider at least the main known interactions present in this symbiosis to obtain more realistic results.

## 6 Conclusion and perspectives

Between the late 1990s and early 2000s, *Escovopsis* emerged as an important mycoparasite of the fungus garden of this complex system of the attine ants. As illustrated above, it is only in the last decade or so that we are actually beginning to define members of the clade with full and robust taxonomic descriptions, now using standardised criteria (Montoya et al., 2021, 2023). A peculiarity of the group is the considerable variation in morphology between members. Morphological characterisation of species is indispensable to their classification, but it can likewise be fundamental to give indications about their relationship with the host fungus and strategy for its exploitation. Conidia of *Escovopsis moelleri*, for example, are larger (approx. 10 µm in length) than those observed in other species and present a distinct apical cap-like structure (Augustin et al., 2013). Can this sort of feature be related to ecological function (e.g. mycoparasitism, dormancy, dispersal etc)? Similarly, other aspects such as dormancy, production of soluble and volatile compounds and growth rate might give us clues about the strategies used by members of the group.

Although there are divergences, *Escovopsis* has characteristics also observed in other mycoparasitic fungi, such as its closest relatives (e.g. *Trichoderma*). Studies of these characteristics have predominantly focused on two species to date: *E. weberi* – the vast majority of studies – and *E. moelleri* – (de Mendonça et al., 2021), in addition to other isolates not formally described. Among the latter, there has been a wealth of studies matching isolates of *Escovopsis* with their hosts *in vitro*, examining inhibition of one by the other, growth of one towards the other and the secondary metabolites that may mediate these interactions. The volatile profiles of *L. gongylophorus* and *Escovopsis* can be useful for future surveys involving specificity in the relationship between these two fungi from different species of attines or even as additional tools for taxonomic and phylogenetic studies (see Croxatto et al., 2012).

The evolutionary history of the group is still under examination, in particular how much of this history is or is not congruent with those of the ant and basidiomycete hosts. There are other questions related to the group’s evolutionary history, such as whether endophytism has played a role. The *Hypocreales* are a fascinating group with a history of switching lifestyles between animal, plant and fungal hosts and in many cases of retaining a capacity to infect more than one of these. As discussed above, much of what we know of the *Escovopsis* group has come from restricted geographical regions. More comprehensive sampling of the tropical and subtropical Americas will be informative and it would be fascinating to look at the mycoparasites of hosts that have restricted ranges and are isolated by geographical barriers.

The mystery of how *Escovopsis* is transmitted between colonies also remains to be resolved. Taxonomic and phylogenetic surveys can be useful here. It is clear that trying to shed light on the life cycle of *Escovopsis* using one species of one genus is like understanding a puzzle of a panoramic photo using pieces of one pixel at a time. Next to nothing is known about its transmission between colonies – the one study that may have shown the first steps of transmission (Augustin et al., 2017) was with a species with external waste dumps, but this is the exception rather than the rule - there are few species of higher attines that have waste external to the colony, for example. There may be evidence that conidia could be phoretic – more work on this could come from rearing experiments coupled with advanced imaging to show if *Escovopsis* conidia are consistently found on ant or inquiline integuments, and from there whether this can lead to new infections of colonies. Similarly, the morphology of members of the group can be compared with the way in which they reach new colonies: are there any that can be associated with plants carried by ants? Which of them are possibly transmitted by other insects and other small arthropods phoretically? There are many questions that still need further investigation.

Considering the entirety of this review, and given the information available at present, we have concluded here that at least some strains of *Escovopsis weberi* are indeed mycoparasites of their basidiomycete hosts in terms of the direct interaction between the two. However, since the presence of mechanisms for parasitism are completely unknown for the vast majority of the *Escovopsis* species (for 24 out of the 25 formally described species), we concluded that caution is advised to consider the entire genus as a mycoparasite. At the colony level, however, it is probably a parasite with a very low virulence and/or an opportunist that is sitting and waiting to overgrow a weakened nest and then effect transmission. The *Escovopsis* – fungal cultivar – ant interaction occurs only in nests of the *Attina* subtribe and it seems that *Escovopsis* is unable to infect and overtake the entire system under normal conditions. *Pseudoxylaria*, a genus associated with fungus-growing termites, has a strategy similar to that which we propose for *Escovopsis* (Visser et al., 2011). Even though present in termite nests, *Pseudoxylaria* species are imperceptible until the activity of termites is reduced for some external reason such as death of the queen or presence of entomopathogenic fungi, allowing *Pseudoxylaria* to overgrow the fungus cultivated by termites. There is thus a precedent for the idea that *Escovopsis* is of low virulence or commensal. Testing this at the colony level is unlikely to be feasible, unless attines with smaller colonies are used – in this case, the ideal proxy for fitness would be the production of alates.

The taxonomic uncertainty that has plagued studies with *Escovopsis* has impacted efforts to evaluate its virulence, as it is not really known if findings are applicable to the entire clade. Ultimately, the strategy used by a given *Escovopsis* species, including its virulence, is dependent on the species or isolate. We know that *Escovopsioides*, for example, is an antagonist of fungi cultivated by ants, but is not aggressive when compared to *Escovopsis in vitro* (Varanda-Haifig et al., 2017; de Mendonça et al., 2021; Pietrobon et al., 2022). However, we have little additional information about *Escovopsioides*. Future surveys should reveal much of the diversity of these fungi, including critical information about their transmission, levels of virulence, the nature of the interactions they establish and their evolution within the attine ant system. Therefore, we emphasise the importance of taxonomic and phylogenetic studies so that the clade is delimited and inferences about the ecological role of *Escovopsis* are more assertive. This gives a structure or context within which studies on different members of the group can be developed. Thus, future research could evaluate the parasitic nature of new species, in particular comparing the strategies of the morphologically different isolates. Likewise, it should be possible to compare isolates that are phylogenetically more closely related to those that are more distant.

As we noted at the outset, leafcutters are major pests of agriculture and silviculture in the Americas. This might lead one to ask what the potential of *Escovopsis* and its allies is as potential biological control agents. We have argued that this group of fungi is not especially virulent, especially against established colonies. Even notable entomopathogenic fungi such as *Metarhizium* spp. and *Beauveria* spp. have not yet been developed as effective biocontrol agents against these insects, so our opinion is that this would be all the more difficult for *Escovopsis*. The need for novel means of control of these pest insects is pressing, however, and there are indeed efforts to develop *Escovopsis* as a biocontrol agent (Queiroz et al., 2024). It seems possible, therefore, that some innovative strategy could be developed. It would probably need to be allied with a strategy to debilitate the colony’s (quite comprehensive) defences such that the antagonistic fungi could take hold and damage or kill the colony. Finally, the subtribe *Attina*, ants that grow fungus as a nutritional source, live in an environment rich with symbiotic interactions, as observed in other fungus-growing insects. Some of these relationships are well-studied, but there are probably countless other relationships of which we are not even yet aware, which may even influence the interactions already established. This promises to be an area of great interest in the future.
